# Anthropometrical measurements and maternal visceral fat during first half of pregnancy: a cross-sectional survey

**DOI:** 10.1186/s12884-020-03258-3

**Published:** 2020-09-29

**Authors:** Daniela Cortés Kretzer, Salete Matos, Lisia Von Diemen, José Antônio de Azevedo Magalhães, Alice Carvalhal Schöffel, Marcelo Zubaran Goldani, Alexandre da Silva Rocha, Juliana Rombaldi Bernardi

**Affiliations:** 1 Faculty of Medicine, Postgraduate Program in Child and Adolescent Health, Universidade Federal do Rio Grande do Sul (UFRGS), Rua Ramiro Barcelos, 2400, Santa Cecilia, Rio Grande do Sul 90035-003 Porto Alegre, Brazil; 2 Postgraduate Program in Psychiatry and Behavioral Sciences, Universidade Federal do Rio Grande do Sul (UFRGS), 90035-003 Porto Alegre, Brazil; 3 Maternal-Fetal Division (Head), Hospital de Clínicas de Porto Alegre, School of Medicine, Federal University of Rio Grande do Sul, Porto Alegre, Brazil; 4 Department of Social and Behavioural Health Sciences, Dalla Lana School of Public Health, University of Toronto, Toronto, Canada; 5 Department of Pediatrics, Hospital de Clínicas de Porto Alegre, School of Medicine, Federal University of Rio Grande do Sul, Porto Alegre, Brazil; 6 Department of Nutrition, Graduate program in Child and Adolescent Health and Graduate Program in Food, Nutrition and Health, Hospital de Clínicas de Porto Alegre, School of Medicine, Federal University of Rio Grande do Sul, Porto Alegre, Brazil

**Keywords:** Anthropometry, Pregnant women, Mid-upper arm circumference, Body mass index

## Abstract

**Background:**

Determining anthropometric measures that indicate different fat deposits can be useful to predict metabolic risk and set specific treatment goals, reducing negative consequences for maternal and fetal health. In cases where pre-gestational weight measure and subsequent body mass index (BMI) values cannot be determined, other anthropometric measurements may be ideal for measuring the nutritional status of pregnant women, especially in low- and middle-income countries. This study aims to identify which anthropometric measurements correlate better with the maternal fat deposits measured by ultrasound.

**Methods:**

A cross-sectional study was conducted with pregnant women from the city of Porto Alegre (city), capital of Rio Grande do Sul (state), southern Brazil, from October 2016 until January 2018. Anthropometrical variables (weight, height, mid-upper arm circumference [MUAC], circumferences of calf and neck and triceps skinfolds [TSF] and subscapular skinfolds [SBSF]), and ultrasound variables (visceral adipose tissue [VAT] and total adipose tissue [TAT]) were collected. To verify the correlation of anthropometric and ultrasound measurements, a non-adjusted and adjusted Spearman correlation was used. The study was approved by the ethics committees.

**Results:**

The age median of the 149 pregnant women was 25 years [21–31], pre-pregnancy BMI was 26.22 kg/m² [22.16–31.21] and gestational age was 16.2 weeks [13.05–18.10]. The best measurements correlated with VAT and TAT were MUAC and SBSF, both of which showed a higher correlation than pre-pregnancy BMI.

**Conclusions:**

It is possible to provide a practical and reliable estimate of VAT and TAT from the anthropometric evaluation (MUAC or SBSF) that is low cost, efficient and replicable in an outpatient clinic environment, especially in low- and middle-income countries.

## Background

Physiological adaptations during pregnancy are caused in order to ensure an adequate supply of nutrients to the fetus [1]. Among these adaptations, the accumulation of fat in different deposits is associated with metabolic consequences for the gestational environment. The mechanisms responsible for the structural and functional differences specific to adipose tissue deposits are still being investigated [2]. However, it is known that visceral fat is strongly associated with increased metabolic diseases [3]. The investigation of measures that assist in the identification of different fat deposits can be useful to predict risk and set specific treatment goals, reducing negative consequences for fetal and maternal health. The prenatal care is an opportune time, as it is characterized by a preventive practice recommended during pregnancy that provides a perspective for important healthcare functions, including health promotion, screening and diagnosis, and disease prevention [4].

For the mother’s anthropometry, the pre-pregnancy body mass index (BMI) is considered a reflection of maternal nutritional status before pregnancy, while gestational weight gain is the aggregate change of the mother’s, child’s and placental mass in the physiologic state [5, 6]. Utilizing BMI as a measurement of health during pregnancy can have limitations, primarily due to pregnancy-associated weight gain and oedema, as well as late booking into antenatal care in a population-level setting [6]. The assessment of the amount of maternal fat deposits during pregnancy is limited by the inability to use ionizing radiation in computerized tomography and dual-energy x-ray absorptiometry, high cost and maintenance of nuclear magnetic resonance and low accuracy of bioelectric impedance analysis. Thereafter, the most commonly used method to measure maternal body composition changes in pregnancy is anthropometry, particularly the use of skinfolds and circumferences [1, 7].

The use of ultrasound to measure the distribution of maternal visceral adipose tissue (VAT) and total adipose tissue (TAT) is becoming useful because it is widely available in hospitals to predict a higher risk of preeclampsia [8], insulin resistance and metabolic diseases [9, 10], premature birth [8] and average birth weight [11]. It is worth mentioning that ultrasound is an easy, quick, safe, non-invasive, precise and reliable method to identify patients with adverse metabolic profiles [12]. However, due to the easiness of execution and low cost, anthropometrical measurements, like mid-upper arm circumference (MUAC), may become an alternative to the use of ultrasound devices [6, 13–15].

There were no found studies that determined the predictive capabilities of anthropometric measures alternative to pre-pregnancy BMI in relation to a reference method, such as VAT and TAT obtained by ultrasound. The aim of this study is to identify which anthropometric measurements correlate better with the maternal fat deposits measured by ultrasound.

## Methods

### Design

The cross-sectional study recruited patients from 2016 to 2018 at the Ultrasound Department of Murialdo Health Center School that provides services of fetal medicine to Brazil’s Unified Health System at the city of Porto Alegre, Rio Grande do Sul, Brazil.

### Participants

For this study, single pregnancies, below twenty gestational weeks, with no evident fetal malformations, with no scars in the abdominal cavity or in sites to use adipometer that hide the measurements and scheduled for routine appointments in Brazil’s Unified Health System were included. The pregnant women who met the inclusion criteria were invited to participate and, after informed consent, were included with the completion of the maternal, clinical and epidemiological questionnaire.

### Measures

The maternal anthropometric evaluation included assessment of anthropometric measures (weight and height) and evaluation of body composition. The participants were encouraged to use minimal clothing and no shoes and accessories like watches, bracelets and earrings. The body weight was measured in kilograms with portable electronic digital scale Marte® LC200-PP (São Paulo, São Paulo, Brazil) accurate to 50 g. The height was measured in meters with extensible portable stadiometer Alturexata® (Belo Horizonte, Minas Gerais, Brazil). Maternal pre-pregnancy weight was collected from the prenatal pregnant chart and confirmed by the maternal report and, when there was no record, in the BMI referring to the first trimester of pregnancy. The pre-pregnancy BMI (in kg/m²) was calculated through the formula, current weight divided by the current height squared. The classification used was pre-pregnancy BMI in underweight (BMI < 18.50 kg/m²), adequate weight (BMI between 18.50 and 24.99 kg/m²), overweight (BMI between 25.00 and 29.99 kg/m²) and obese (BMI ≥ 30.00 kg/m²), according to categories defined by World Health Organization [16].

Perimeters were measured with an anthropometric tape on the right trunk, arm and leg. Calf perimeter was measured at the greater circumference. The neck perimeter was measured at the midpoint between the clavicle bone and chin. MUAC was measured at the midpoint between the acromion and olecranon bones. Triceps (TSF) and subscapular skinfolds (SBSF) were evaluated using a caliper (Lange®). The TSF was measured at the same levels as those of arm perimeter. The SBSF was measured two centimeters below the lower angle of the scapula bone. Anthropometric data were measured in duplicate by nutritionists, considering the arithmetic mean value among the measurements.

Measurement of maternal abdominal fat space was done with ultrasound device Toshiba Xario XG® with a 3.5 mHz multi-frequency convex probe placed above the maternal umbilical scar, with low pressure, and automatic calipers were positioned from anterior aortic wall to linea alba measuring maternal abdominal depth. Two measurements were performed by only one specialist medical researcher, first during maternal inspiration and after during maternal expiration. The arithmetic mean of measurements was used for this research. The measurement of maternal subcutaneous adipose tissue (SAT) was made in the same position as that of VAT measurement, with the automatic caliper positioned from linea alba to dermal edge on the surface of the maternal abdomen. The sum of VAT and SAT was used to estimate the total adipose tissue (TAT) during the evaluation.

### Statistical analysis

Statistical analyses were performed through Statistical Package for Social Sciences (SPSS) 21.0. The level of significance was set at *p* < 0.05. Clinical, epidemiological and ultrasonographic data were presented as quantitative and categorical variables. The test of normality of variables distribution was made. Quantitative variables with asymmetric distributions were described as median and interquartile range. Categorical variables were reported as absolute frequency and percentage. To perform the associations between anthropometrical measurements and VAT and TAT measurements, non-adjusted Spearman correlation (*ρ*) was used. After that, Spearman correlation was adjusted for factors associated with maternal adipose accumulation during pregnancy: number of pregnancies, pregnant age and gestational age.

### Ethical Aspects

The study was approved by the Research Ethics Committees of the Health Department of Porto Alegre under number 2.132.090 and in the Presidente Vargas Hospital under number 1.758.959. Written informed consent was obtained from participants.

## Results

The sample was comprised of 149 pregnant women up to 20 weeks of pregnancy. Nineteen participants were not included in the correlation analysis of the study outcomes due to the decision not to include cases that had some unanswered adjustment variable. Participants were on median 25 years of age [21–31], mostly Caucasian (54.8%, n = 80), had a median pre-pregnancy BMI of 26.22 kg/m² [22.16–31.21], 58.33% (n = 84) with pre-pregnancy BMI classified as overweight or obesity, often had two past pregnancies [1 - 3] and a gestational age average of 16.2 weeks [13.05–18.10]. The anthropometric measurements showed that the MUAC median was 31.0 cm [28.0–35.0], calf perimeter median was 37.0 cm [35.0–41.0] and neck perimeter was 34.0 cm [32.0–36.0]. TSF and SBSF showed median of 31.5 mm [25.0–40.5] and 30.0 mm [21.0–40.5], respectively. The median VAT was 41.7 mm [34.5–52.8] and TAT was 61.1 mm [50.72–71.42], as shown in Table 1.

**Table 1:** Maternal demographic, gestational and clinical characteristics

Variables	n (%)	Median (IQ)
**Age (years)**	149	25.00 [21.00–31.00]
**Number of pregnancies**	143	02 [01–03]
**Gestational age (weeks)**	149	16.20 [13.05–18.10]
**Race** **White** **Black** **Dark-skinned**	146 80 (54.80) 42 (28.80) 24 (16.40)	
**Pre-pregnancy body mass index (kg/m²)**	144	26.22 [22.16–31.21]
**Underweight**	3 (2.08)	
**Adequate**	57 (39.58)	
**Overweight**	37 (25.70)	
**Obesity**	47 (32.64)	
**Arm circumference (cm)**	147	31.00 [28.00–35.00]
**Neck circumference (cm)**	147	34.00 [32.00–36.00]
**Calf circumference (cm)**	146	37.00 [35.00–41.00]
**Triceps Skinfold (mm)**	147	31.50 [25.00–40.50]
**Subscapular Skinfold (mm)**	147	30.00 [21.00–40.50]
**Central Visceral Fat (mm)**	149	41.70 [34.50–52.80]
**Total Central Fat (mm)**	142	61.20 [50.65–71.75]

Descriptive table with medians [interquartile interval] and frequency (%)

Totals may not add up to 149 because of missing values

Table 2 shows the non-adjusted correlation across anthropometrical measurements and pre-pregnancy BMI values with ultrasound measurements of the maternal abdomen. We can observe that all body perimeters and skin folds showed to be statistically correlated to VAT and TAT. When analyzed individually, calf and neck perimeters and TSF indicate weaker correlations to detect VAT and TAT, when compared to pre-pregnancy BMI. However, MUAC and SBSF presented greater correlations with VAT and TAT, when compared to pre-pregnancy BMI.

**Table 2:** Non-adjusted correlation of anthropometrical measurements and pre-pregnancy body mass index values with ultrasound measurements of maternal abdomen

Variables		Mid-upper arm circumference (cm)	Calf circumference (cm)	Neck circumference (cm)	Triceps Skinfold (mm)	Subscapular Skinfold (mm)	Pre-pregnancy BMI (Kg/m²)
**Central Visceral Fat** **(mm)**	*ρ*	0.603*	0.498*	0.500*	0.541*	0.597*	0.546*
	n	147	146	147	147	147	144
**Total Central** **Fat (mm)**	*ρ*	0.792*	0.677*	0.656*	0.698*	0.740*	0.725*
	n	140	139	140	140	140	137

*BMI *Body mass index

Spearman correlation; *p value < 0.05

Totals may not add up to 149 because of missing values

Table 3 presents the adjusted correlation between anthropometric measurements, pre-pregnancy BMI with ultrasound measurements of the maternal abdomen. It is worth mentioning that even with the adjustment for variables associated to maternal adipose accumulation during pregnancy (i.e., number of pregnancies, maternal age and gestational age), the measurements of MUAC, TSF and SBSF maintained statistical significance (*p* < 0.05), showing values higher than pre-pregnancy BMI.

**Table 3:** Adjusted correlation of anthropometrical measurements and pre-pregnancy body mass index values with ultrasound measurements of maternal abdomen

Variables		Control Variables	Mid-upper arm circumference (cm)	Triceps Skinfold (mm)	Subscapular Skinfold (mm)	Pre-pregnancy BMI (Kg/m²)
***Central Visceral Fat*(mm)**	Coef. ρ n = 130	Without adjustment	0.603*	0.541*	0.597*	0.546*
		Number of **pregnancies** + Maternal age (years) + Gestational age (weeks)	0.598*	0.598*	0.602*	0.543*
***Total Central Fat*(mm)**	Coef. ρ n = 130	Without adjustment	0.792*	0.698*	0.740*	0.725*
		Number of **pregnancies** + Maternal age (years) + Gestational age (weeks)	0.752	0.712	0.719	0.691*

*BMI* Body mass index

Spearman correlation adjusted to number of children, maternal age and gestational age. *p value < 0.05

Totals may not add up to 149 because of missing values

## Discussion

The study found that the MUAC and SBSF measures presented greater correlations with VAT and TAT during the first 20 weeks of pregnancy, with a higher correlation than the pre-pregnancy BMI value. It is important to emphasize that these anthropometric measurements are considered low cost, efficient and replicable in in under-resourced settings.

These findings are particularly valuable in cases where pregnancy discovered late or if the individual does not accurately remember their pre-gestational weight in clinical practice, making it difficult to correctly estimate pre-pregnancy BMI. Currently, pre-pregnancy BMI is the anthropometrical indicator of the nutritional state most used as a metabolic risk marker, because women who were overweight or obese are at an elevated relative risk of preeclampsia [17, 18], cesarean section delivery [17], gestational diabetes [18], increasing the relative risk of intrauterine death [18] and more likely to be macrosomic [17]. However, this index is limited with regard to the differentiation of adipose content [1], particularly in the central region, the focus of the present study. Furthermore, despite the ease of measuring BMI, it has low predictive precision for abnormal pregnancy results; therefore, new diagnostic modalities can improve these scores, as demonstrated by Bourdages et al. (2018) and Souza et al. (2016), where the increase in visceral adipose tissue, identified during the first trimester, was associated with a greater chance of developing gestational diabetes mellitus [9, 19].

Research indicates that the use of circumferences and skinfolds to determine maternal nutritional state during the first weeks of pregnancy can facilitate metabolic risk detection [1, 7]. A study made in Nigeria with 578 pregnant women showed that MUAC has a strong positive correlation with maternal weight and could be used to identify obesity in women regardless of stage of pregnancy. The authors found that MUAC values of 33 cm might be reliable cut off points for diagnoses of obesity throughout pregnancy [15]. Another study in Central Malaysia with 498 pregnant women found that MUAC was inversely associated with an inadequate rate of gestational weight gain, as compared to normal gestational weight gain [13]. Besides that, a cross-sectional study conducted in South Africa with 164 women showed a strong correlation between MUAC and pre-pregnancy BMI in pregnant women up to 30 weeks’ gestation. The authors found that the MUAC cut-offs for obesity and malnutrition were calculated as 30.57 cm and 22.8 cm, respectively [6].

Along with the use of MUAC to determine maternal metabolic risk, SBSF proved useful in detecting low weight newborns in a prospective study conducted in Argentina with 488 pregnant women. The authors found that a low increase of skinfolds during pregnancy can indicate low birthweight, demonstrating significant consequences to the offspring’s health [20, 21].

On the other hand, the measurement of the different compartments of abdominal fat via ultrasound provides an adequate estimate of central adiposity [22]; however, the assessment of maternal central fat is not routinely performed in obstetric ultrasounds. The risk of adverse conditions caused by an excess of fat, particularly visceral fat, to the pregnant woman and fetus health is clearly consolidated in the literature [8, 10, 17, 23, 24], therefore, thus the precision and cost appraisal of different fat compartments is highly important to the population [25].

Aligned with the Brazilian Ministry of Health recommendations on resolute prenatal care [26] and the isolated capacity of MUAC and SBSF to detect the increase in amounts of maternal central fat, the inclusion of clinical anthropometry during the first 20 weeks of pregnancy can contribute to accurate maternal metabolic risk prediction. Thus, their complementary use in clinical practice is justified, as well as their possible inclusion in protocols for nutritional assessment during pregnancy. MUAC in particular can act as an alternative tool in screening for patients with metabolic risk in developing countries where monitoring of weight gain is not feasible due to limitations involving equipment (adipometer, for example), team or prenatal coverage.

It is well known that the early diagnosis of metabolic risk during the first half of pregnancy allows for the early implementation of preventive and therapeutic measures, resulting in improved maternal and fetal health due to immediate action following diagnosis [27]. On the other hand, cases where the maternal central fat estimate is appropriate, according to the suggested anthropometry, even among obese women, the high cost of following patients without metabolic risk and high risk prenatal could be avoided.

### Strengths and limitations

The study was conducted in a low risk primary healthcare setting that did not provide additional intervention to the sample. The data were obtained from routine follow-up of patients, which suggests that the findings can be easily transferred to the clinical practice. The most important aspect of the research was that the study’s population was comprised of pregnant women where the anthropometrical measurements can be become tools for decision making in clinical practice both in low and high obstetric risk environments. The low number of researchers involved in data collection minimizes possible mistakes of measurement. Throughout the study, one researcher was in charge of ultrasound collections and two researchers were in charge of the general questionnaire and nutritional assessment. A limitation of the study is the cross-sectional design that prevents the verification of pregnancy outcomes among women with large amounts of central fat due to the absence of blood sampling that diagnoses metabolic risk in pregnancy. Another limitation of the study includes the self-reporting of pre-pregnancy weight, as it may have been affected by recall bias. However, several studies demonstrated that the use of self-reported weight in pregnant women and young adults is valid [28, 29].

## Conclusions

The study found that the anthropometric measurements most correlated with VAT and TAT were MUAC and SBSF, both of which had a higher correlation than pre-pregnancy BMI. It is possible to provide a practical, reliable and low-cost clinical estimate of ultrasound measurements of maternal central fat, which will help to identify women at high metabolic risk during pregnancy, based on efficient and replicable anthropometrical examination. In situations where pre-gestational weight measurement and subsequent BMI values cannot be obtained, the anthropometric measurements, MUAC and SBSF are useful and may be ideal for measuring the nutritional status of pregnant women, especially in low- and middle-income countries.

## Abbreviations

BMI: Body mass index
HOMA-IR: Homeostasis model assessment
MUAC: Mid-upper arm circumference
SAT: Subcutaneous adipose tissue
SBSF: Subscapular skin folds
SPSS: Statistical package for social sciences
TAT: Total adipose tissue
TSF: Triceps skin folds
VAT: Visceral adipose tissue

## Availability of data and materials

The datasets generated and/or analyzed during the current study are not publicly available due to patient confidentiality reasons as well as the use of the same data for the development of other manuscripts.
